# Polymyxin B-Associated Nephrotoxicity and Its Predictors: A Retrospective Study in Carbapenem-Resistant Gram-Negative Bacterial Infections

**DOI:** 10.3389/fphar.2022.672543

**Published:** 2022-04-28

**Authors:** Xiao-Li Wu, Wen-Ming Long, Qiong Lu, Xin-Qi Teng, Ting-Ting Qi, Qiang Qu, Ge-Fei He, Jian Qu

**Affiliations:** ^1^ Department of Pharmacy, The Second Xiangya Hospital, Institute of Clinical Pharmacy, Central South University, Changsha, China; ^2^ Department of Pharmacy, The Second Affiliated Hospital of Guangzhou Medical University, Guangzhou, China; ^3^ Department of Pharmacy, Second People’s Hospital of Huaihua City, Huaihua, China; ^4^ Department of Pharmacy, Xiangya Hospital, Central South University, Changsha, China; ^5^ Department of Pharmacy, The First Hospital of Changsha, Changsha, China

**Keywords:** polymyxin B, nephrotoxicity, acute kidney injury, carbapenem-resistant Gram-negative bacteria, adverse reactions

## Abstract

Polymyxin B (PMB), a kind of polymyxin, was widely used in carbapenem-resistant Gram-negative bacterial (CR-GNB) infections. However, adverse reactions such as nephrotoxicity and neurotoxicity limit its use in clinical practice. The aim of this study was to explore PMB associated with nephrotoxicity and its predictors. Patients who received PMB intravenous drip for more than 72 h were eligible for the study. Characteristics of patients, concomitant nephrotoxic agents, underlying disease, and antimicrobial susceptibility were submitted for descriptive analysis. Univariate analysis and binary logistic regression were used to assess the factors leading to acute kidney injury (AKI). AKI was assessed with serum creatinine variations according to the classification of risk (stage R), injury (stage I), failure (stage F), loss, and end-stage of kidney disease. Among 234 patients with CR-GNB infections who used PMB in our study, 67 (28.63%) patients developed AKI, including 31 (14.25%) patients in stage R, 15 (6.41%) patients in stage I, and 21 (8.97%) patients in stage F. The incident rate of PMB-related nephrotoxicity in patients with normal renal function was 32.82% (43/131). The higher risk factors of AKI include males [odds ratio (OR) = 3.237; 95% confidence interval (95%CI) = 1.426–7.350], digestive system diseases [OR = 2.481 (1.127–5.463)], using furosemide (>20 mg/day) [OR = 2.473 (1.102–5.551)], and baseline serum creatinine [OR = 0.994 (0.990–0.999)]. Nonparametric tests of K-independent samples showed that baseline serum creatinine and the PMB maintenance dose were associated with the severity of nephrotoxicity (both *p* < 0.05). Male, digestive system diseases, using furosemide (>20 mg/day), and high baseline serum creatinine were the independent risk factors of PMB-associated AKI development. The maintenance dose of PMB may be related to the severity of AKI. These risk factors should be taken into consideration when initiating PMB-based therapy. The serum creatinine value should be closely monitored when using PMB.

## Introduction

Due to nephrotoxicity concerns, the clinical use of polymyxins was considerably reduced in the early 1970s (Keirstead et al., 2014). The nephrotoxicity rates of polymyxins reported range from 11.8 to 58.1% (John et al., 2017; Aggarwal and Dewan, 2018; Yarelis et al., 2019; Sisay et al., 2020). However, polymyxin was reintroduced in clinical practices worldwide because of the increasing incidence of infections caused by multi-drug resistant (MDR) Gram-negative microorganisms and the lack of new antibiotics. Multi-drug resistant (MDR) bacteria are defined as the bacteria that exhibited resistance to clinically used class 3 or more antimicrobial drugs simultaneously. PMB has been widely used as a kind of polymyxin in carbapenem-resistant Gram-negative bacterial (CR-GNB) infections (Vaara, 2019). Because PMB was developed before modern drug development trials, there is little data available on its pharmacokinetics and pharmacodynamic properties. Increasing pharmacokinetics and pharmacodynamics studies on PMB have been published, providing a more precise dosing strategy to maximize the efficacy and reduce nephrotoxicity (Quagliano et al., 2020; Wang et al., 2020; Xie et al., 2020).

Although polymyxins showed strong antimicrobial activity against Gram-negative bacteria, their adverse reactions (ADRs), such as nephrotoxicity and neurotoxicity, limit their clinical use (Soman et al., 2020). Pierson-Marchandise et al. (2016) analyzed 38,782 ADRs recorded in the French national pharmacovigilance database and found that 3.2% of ADRs were classified as cases of acute kidney injury (AKI). AKI increases the risk of death and serious morbidity in hospitalized patients (Hoste et al., 2015). Previous studies investigated the nephrotoxicity of PMB and colistin and the risk factors of polymyxin-associated AKI (Aggarwal and Dewan, 2018; Ozel et al., 2019; Almutairy et al., 2020; Cai et al., 2020; Zhang et al., 2021). Moreover, some system reviews and meta-analyses focused on the nephrotoxicity of polymyxins. They found that malignancy, co-infection with other microorganisms, being elderly, the high daily dose of PMB, having underlying diseases such as diabetes, and use of concomitant nephrotoxic drugs were independent predictors of nephrotoxicity (Oliota et al., 2019; Sisay et al., 2020; Wagenlehner et al., 2020). Ritesh et al. compared the nephrotoxicity of colistin with PMB administered in currently recommended doses and found that PMB is less nephrotoxic than colistin (Aggarwal and Dewan, 2018). However, there is only one amino acid difference between PMB and colistin, which had been documented to have well-known nephrotoxicity and neurotoxicity (Zavascki and Nation, 2017). The relative safety of the two agents requires closer examination in well-designed clinical studies (Zavascki and Nation, 2017).

Studies have demonstrated that PMB is substantially accumulated in proximal renal tubules and potentially toxic to renal tubular cells in *in vitro* and *in vivo* models (Zavascki and Nation, 2017; Azad et al., 2019). Cai et al. (2020) found that higher daily PMB doses (∼30,000 IU/kg/day) and the higher number of concurrent nephrotoxins were independently associated with AKI. Despite previous reports on the incidence of PMB-related nephrotoxicity ranging from 14.0 to 50.6% and the associated risk factors (Nelson et al., 2015; Soares et al., 2017; Cai et al., 2020; Sisay et al., 2020; Zhang et al., 2021), the association between PMB and the development of nephrotoxicity remain inconclusive, especially the dosing and concurrent nephrotoxins and underlying diseases such as kidney disease. Moreover, considering the racial differences in drug dose and PK/PD, it is imperative to explore clinical variables that affect the nephrotoxicity of PMB among Chinese patients.

In this study, we aim to evaluate the nephrotoxicity of Chinese patients using PMB and the possible risk factors for AKI caused by PMB to provide a reference for clinical use of PMB.

## Patients and Methods

### Ethics

Our study was approved by the Ethics Committees of The Second Xiangya Hospital of Central South University (LYF-2020021) on 2 September 2020. We followed the ethical standards laid down in the 1964 Declaration of Helsinki. Because of the non-interventional nature of our study, informed consent was waived.

### Patients

We conducted a retrospective cohort study in The Second Xiangya Hospital of Central South University from 1 May 2018 to 28 February 2020. We included adult (>18 years of age) patients who received PMB (Shanghai Number 1 Biochemical and Pharmaceuticals, Shanghai, China) intravenous drip for more than 72 h and were infected with CR-GNB. The exclusion criteria were: 1) < 18 years old or pregnant and 2) incomplete clinical data.

### Clinical Data Collection

Clinical data extracted from patients’ electronic records included demographics such as age and sex, comorbidities, type of infection and CR-GNB, Acute Physiology and Chronic Health Evaluation II (APACHE II) score at the time of CR-GNB infection, infection sites, details of PMB use (loading dose, daily dose based on total body weight, duration of treatment, and cumulative PMB dose), concomitant nephrotoxic agents, and pre-medication kidney disease.

### Outcome Definitions

The primary outcome was that any stage of AKI occurs after 48 h from the use of PMB. According to the Kidney Disease: Improving Global Outcomes (KDIGO) criteria (Kdigo and Outcomes, 2012), AKI was defined as an increase in serum creatinine by 0.3 mg/dl within 48 h or by a 50% increase in serum creatinine within 7 days. Acute kidney disease (AKD) was defined as GFR <60 ml/min per 1.73 m^2^ for <3 months (Levey et al., 2020). The criteria of chronic kidney disease (CKD) was GFR <60 ml/min per 1.73 m^2^ for >3 months (Levey et al., 2020). Normal renal function was considered GFR ≥60 ml/min per 1.73 m^2^. We did not assess urine output changes because urine output documentation is less reliable retrospectively, and drug-induced AKI typically does not produce oliguria. Renal function was assessed with serum creatinine variations according to the classification of risk, injury, failure, loss, and end-stage of kidney disease (RIFLE) (Bellomo et al., 2004). The maximum stages of AKI were determined with blood creatinine levels within 48 h of discontinuation of PMB, which were 1.5–1.9 times, 2.0–2.9 times, and three times higher than the baseline and defined as AKI-phased R, I, and F. Baseline creatinine was the last creatinine obtained before antibiotic initiation (as previously stated, this value must have been obtained within 48 h of antibiotic initiation). Follow-up continued until 48 h after the last dose. Diseases of the digestive system include gastroenteritis, gastrointestinal hemorrhage, pancreatitis, and choledocholithiasis.

### Statistical Analysis

All data were analyzed using SPSS 25 (IBM, Armonk, NY, United States). Continuous data were represented by mean and standard deviation (SD) or median and interquartile ranges (IQRs). Count data were presented as absolute numbers and percentages. To determine the factors associated with AKI in this cohort study, univariate analysis and binary logistic regression were used to compare patients with and without AKI. Factors with *p*-values < 0.1 by univariate analysis were entered into the multivariate logistic analysis. Nonparametric tests of K-independent samples were used to investigate the risk factors associated with AKI severity in patients using PMB. A final two-tailed *p*-value less than 0.05 was considered significant.

## Results

### Patient Characteristics

**FIGURE 1:**
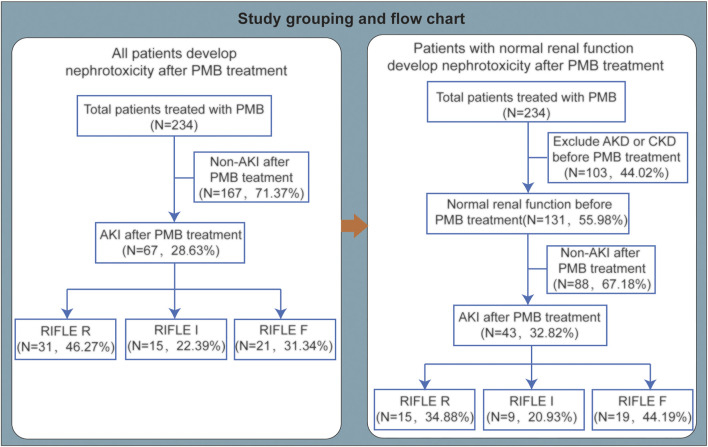
Study grouping and flow chart. PMB, polymyxin B; AKI, acute kidney injury; AKD, acute kidney disease; CKD, chronic kidney disease.

According to the inclusion and exclusion criteria, 234 CR-GNB patients were enrolled in our study. The grouping and comparison of enrolled patients are shown in Figure 1. The patient’s baseline demographic and clinical characteristics are shown in Table 1. The median age of the patients was 57.5 years (IQR, 47–70.25 years), and 70.94% of the patients were men. The patients with comorbidities include respiratory disease (84.62%) and cardiovascular and cerebrovascular diseases (62.82%). During hospitalization, 162 (69.23%) patients were admitted to the intensive care unit, and 164 (70.09%) patients received mechanical ventilation. Before using PMB, there were 103 (44.02%) patients who had acute kidney disease (AKD) or chronic kidney disease (CKD) and 131 (55.98%) patients who had a normal renal function. Concomitant nephrotoxic agents include diuretics (26.92%), trimethoprim/sulfamethoxazole (TMP-SMZ) (10.26%), NSAIDs (5.13%), vancomycin (4.27%), amphotericin B (3.85%), aminoglycosides (3.85%), ciclosporin (2.99%), and fluorocytosine (0.85%). The proportion of patients receiving the loading dose (>2 mg/kg) was 20.94%, and the maintenance dose was 50 (50–100) mg or 0.84 (0.83–1.00) mg/kg every 12 h. Only 11.97% of patients had a maintenance dose of more than 1.25 mg/kg every 12 h. The cumulative dose was 950 (600–1,400) mg, and the treatment duration was 9.75 (6.38–13.50) days. The creatinine before administration was 76.7 (47.13–143.05) μmol/L. A total of five patients used continuous renal replacement therapy, and 29 patients used hemodialysis.

**TABLE 1:** Demographics and clinical characteristics of the study cohort.

Clinical characteristic	Normal renal function patients before PMB treatment (*N* = 131)	AKD or CKD patients before PMB treatment (*N* = 103)	Total patients treated with PMB (*N* = 234)	*p*-value∗
Male gender	92 (70.23)	74 (71.84)	166 (70.94)	0.787
Age (years)	56 (48.00–66.00)	61 (46.00–75.00)	57.50 (47.00–70.25)	0.272
Weight (kg)	60.00 (50.00–60.00)	57.00 (50.00–60.00)	60 (50.00–60.00)	0.151
Mechanical ventilation	95 (72.52)	69 (66.99)	164 (70.09)	0.359
Vasoactive agents	68 (51.91)	62 (60.19)	130 (55.56)	0.205
Admission to ICU	98 (74.81)	64 (62.14)	162 (69.23)	**0.037**
APACHE II	17.57 ± 7.98	22.52 ± 8.25	19.59 ± 8.46	**0.000**
Multi-site infection	61 (46.56)	43 (41.75)	104 (44.44)	**0.046**
Site of infection				
*Respiratory tract*	123 (93.89)	97 (94.17)	220 (94.02)	0.928
*Urinary system*	13 (9.92)	14 (13.59)	27 (11.54)	0.383
*Bloodstream*	34 (25.95)	38 (36.89)	72 (30.77)	0.072
*Abdomen*	14 (10.69)	13 (12.62)	27 (11.54)	0.646
Underlying disease				
*Cardiovascular and cerebrovascular diseases*	73 (55.73)	74 (71.84)	147 (62.82)	**0.011**
*Respiratory disease*	112 (85.50)	86 (83.50)	198 (84.62)	0.674
*Liver disease*	39 (29.77)	21 (20.39)	60 (25.64)	0.103
*Diseases of digestive system*	25 (19.08)	18 (17.48)	43 (18.38)	0.753
*Nutritional diseases*	15 (11.45)	13 (12.62)	28 (11.97)	0.784
*Diabetes mellitus*	18 (13.74)	20 (19.42)	38 (16.24)	0.242
Pathogenic bacteria				
*Acinetobacter baumannii*	97 (74.05)	56 (54.37)	153 (65.38)	**0.002**
*Klebsiella pneumoniae*	42 (32.06)	40 (38.83)	82 (35.04)	0.281
*Pseudomonas aeruginosa*	35 (26.72)	29 (28.16)	64 (27.35)	0.807
*Escherichia coli* or *Enterobacter cloacae*	7 (5.34)	3 (2.91)	10 (4.27)	0.361
*Number of CR-GNB (≥2)*	43 (32.82)	21 (20.39)	64 (27.35)	**0.034**
Concomitant nephrotoxic agents				
*Vancomycin*	9 (6.87)	1 (0.97)	10 (4.27)	0.059
*Aminoglycosides*	4 (3.05)	5 (4.85)	9 (3.85)	0.712
*Amphotericin B*	5 (3.82)	4 (3.88)	9 (3.85)	1.000
*TMP-SMZ*	8 (6.11)	16 (15.53)	24 (10.26)	**0.018**
*Fluorocytosine*	1 (0.76)	1 (0.97)	2 (0.85)	1.000
*Furosemide* (>*20 mg/day*)	23 (17.56)	23 (22.33)	46 (19.66)	0.362
*Torasemide* (>*10 mg/day*)	7 (5.34)	10 (9.71)	17 (7.26)	0.202
*NSAIDs*	7 (5.34)	5 (4.85)	12 (5.13)	1.000
*Ciclosporin*	2 (1.53)	5 (4.85)	7 (2.99)	0.273
Antimicrobial susceptibility				
*Polymyxin MIC > 2 (mg/L)*	5 (3.82)	7 (6.80)	12 (5.13)	0.305
PMB use				
*Loading dose (mg)*	50 (50.00–100.00)	50 (50.00–100.00)	50 (50.00–100.00)	**0.000**
*Loading dose (>2 mg/kg)*	31 (23.66)	18 (17.48)	49 (20.94)	0.248
*Maintenance dose q12 h (mg)*	50 (50.00–50.00)	50 (50.00–50.00)	50 (50.00–50.00)	**0.017**
*Maintenance dose in mg/kg(q12h)*	0.83 (0.83–1.00)	0.84 (0.78–1.00)	0.84 (0.83–1.00)	0.229
*Minimum maintenance dose (>1.25 mg/kq, q12 h)*	16 (12.21)	12 (11.65)	28 (11.97)	0.895
*Cumulative dose (mg)*	1,000 (550.00–1,400.00)	950 (640.00–1,400.00)	950 (600–1,400)	0.856
*Treatment duration time (days)*	9.50 (6.00–13.00)	10.00 (7.00–14.00)	9.75 (6.38–13.50)	0.290
Kidney disease-related clinical characteristics#				
*Creatinine before administration(umol/L)*	49.90 (39.00–67.90)	183.30 (104.60–257.80)	76.7 (47.13–143.05)	**0.000**
*CRRT*	1 (0.76)	4 (3.88)	5 (2.14)	0.237
*Hemodialysis*	0 (0)	29 (28.16)	29 (12.39)	**0.000**

Vasoactive agents mainly contain epinephrine, norepinephrine, isopropyl epinephrine, and dopamine during hospitalization; admission to ICU means that the patients were already admitted to the ICU at the time of receiving PMB; ICU, intensive unit care; APACHE II, Acute Physiology and Chronic Health Evaluation II; respiratory tract including bronchial pneumonia, HAP, CAP, and other respiratory infections; CR-GNB, carbapenem-resistant Gram-negative bacteria; TMP-SMZ, trimethoprim/sulfamethoxazole; NSAIDs, nonsteroidal anti-inflammatory drugs; PMB, polyamine B; CRRT, continuous renal replacement therapy; Continuous data were represented as mean ± standard deviation (SD) or median and interquartile ranges (IQRs). Count data were presented as absolute numbers and percentages (%).^∗^ *p*-values were shown as normal renal function patients before PMB treatment vs. AKD or CKD patients before PMB treatment. # denotes patients who may belong to more than one group. Bold font indicates data with significant differences.

Compared with normal renal function patients before PMB treatment and AKD or CKD patients before PMB treatment, we found that admission to ICU, APACHE II, multi-site infection, the underlying disease with cardiovascular and cerebrovascular diseases, *Acinetobacter baumannii* infection, number of CR-GNB (≥2), and a concomitant nephrotoxic agent with sulfamethoxazole were different (all *p* values < 0.05). Renal function such as creatinine before administration was also different between the two groups. Moreover, PMB loading dose (mg) and maintenance dose at q12 h (mg) were different between the two groups (Table 1).

### Incidence and Characteristics of AKI

Among 234 patients with CR-GNB infections who used PMB-based regimens, 67 (28.63%) patients developed AKI, including 31 (46.27%) patients in stage R, 15 (22.39%) patients in stage I, and 21 (31.34%) patients in stage F (Figure 1). Among 67 patients who developed AKI, 65.67% (44 of 67) had no renal insufficiency before PMB use (Table 2). 80.60% of AKI patients were males, and the mean age was 63.96 ± 16.90 years. There were 18 patients with digestive system diseases, and the proportion reached 26.87%. Concomitant nephrotoxic agents in 43% of AKI patients included 29.85% of AKI patients using furosemide (>20 mg/day). 52.24% of AKI patients used a loading dose of 1.33 (0.83–1.67) mg/kg, and there were only 8 (11.94%) patients using loading dose > 2 mg/kg.

**TABLE 2:** Univariate and multivariate analysis for the factors potentially associated with acute kidney injury in patients treated with PMB.

Variable	Univariate analysis			Multivariable analysis	
	AKI [+] (*N* = 67)	AKI [-] (*N* = 167)	*p*-value	Adjusted OR (95%CI)	*p*-value
Male gender	55 (80.60)	112 (67.07)	**0.039**	**3.237 (1.426–7.350)**	**0.005**
Age (years)	63.96 ± 16.90	56.31 ± 16.75	**0.005**	1.015 (0.994–1.036)	0.174
Weight (kg)	60 (53–60)	60 (50–60)	0.199		
Mechanical ventilation	54 (80.60)	11 (65.87)	**0.026**	0.937 (0.366–2.401)	0.892
Vasoactive agents	45 (67.16)	85 (50.90)	**0.024**	1.932 (0.853–4.372)	0.114
Admission to ICU	51 (76.12)	111 (66.47)	0.148		
APACHE II	18.2 ± 8.32	20.40 ± 8.44	0.134		
Multi-site infection	37 (55.22)	67 (40.12)	**0.036**	1.209 (0.554–2.638)	0.633
Site of infection					
*Respiratory tract*	65 (97.01)	155 (92.81)	0.358		
*Urinary system*	6 (8.96)	21 (12.57)	0.433		
*Bloodstream*	26 (38.81)	46 (27.54)	0.092	1.223 (0.529–2.828)	0.637
*Abdomen*	7 (10.45)	20 (11.98)	0.741		
Underlying disease					
*Cardiovascular and cerebrovascular diseases*	41 (61.19)	106 (63.47)	0.744		
*Respiratory disease*	58 (86.57)	140 (83.83)	0.600		
*Kidney disease*	18 (26.87)	59 (35.33)	0.213		
*Liver disease*	18 (26.87)	42 (25.15)	0.786		
*Diseases of digestive system*	18 (26.87)	25 (14.97)	**0.034**	**2.481 (1.127–5.6463)**	**0.024**
*Nutritional diseases*	8 (11.94)	20 (11.97)	0.994		
*T2DM*	11 (16.41)	27 (16.16)	0.960		
Concomitant nephrotoxic agents	30 (43%)	70 (41%)	0.663		
Total number of combined nephrotoxic agents	0 (0–1)	0 (0–1)	0.612		
*Without combined nephrotoxic agents*	37 (55.22)	134 (80.24)	0.689		
*Combined with 1 nephrotoxic agent*	20 (29.85)	50 (29.94)	0.989		
*Combined with 2 nephrotoxic agents*	8 (11.94)	16 (9.58)	0.591		
*Combined with 3 nephrotoxic agents*	2 (4.48)	4 (2.40)	1.000		
*Vancomycin*	0 (0)	10 (5.99)	0.091	0 (0–0)	0.999
*Aminoglycosides*	2 (2.99)	7 (4.19)	0.954		
*Amphotericin B*	1 (1.49)	8 (4.79)	0.418		
*TMP-SMZ*	9 (13.43)	15 (8.98)	0.310		
*Fluorocytosine*	0 (0)	2 (1.20)	1.000		
*Furosemide* (>*20 mg/day*)	20 (29.85)	26 (15.57)	**0.013**	**2.473 (1.102–5.551)**	**0.028**
*Torasemide* (>*10 mg/day*)	6 (8.96)	11 (6.59)	0.725		
*NSAIDs*	3 (4.48)	9 (5.39)	1.000		
*Ciclosporin*	1 (1.49)	6 (3.59)	0.669		
Pre-medication kidney disease	23 (33%)	69 (41%)	0.249		
*CRRT*	1 (1.49)	4 (2.40)	1.000		
*Baseline serum creatinine (umol/L)*	65.20 (40.40–111.60)	81.90 (52.10–189.90)	**0.018**	**0.994 (0.990–0.999)**	**0.008**
*Hemodialysis*	5 (7.46)	24 (14.37)	0.148		
PMB regimen					
*Applied loading dose*	35 (52.24)	65 (38.92)	0.063	2.571 (0.695–9.520)	0.157
*Loading dose in mg/kg*	1.33 (0.83–1.67)	1.00 (0.83–1.67)	0.188		
*Maintenance dose in mg/kg(q12 h)*	0.83 (0.83–1.00)	0.85 (0.81–1.00)	0.609		
*Loading dose(>2 mg/kg)*	8 (11.94)	20 (11.98)	0.994		
*Minimum maintenance dose achieved*	15 (22.39)	34 (20.36)	0.730		
*Cumulative dose*	925 (550–1,650)	975 (600–1,350)	0.719		
*Treatment duration time (days)*	10 (6–13.75)	10 (7–13.5)	0.912		
*Maintenance dose q12 h (mg)*	50 (50–50)	50 (50–50)	**0.048**	0.998 (0.973–1.024)	0.905
*Loading dose (mg)*	80 (50–100)	50 (50–100)	0.057	0.991 (0.970–1.013)	0.417

AKI, acute kidney injury; vasoactive agents mainly contain epinephrine, norepinephrine, isopropyl epinephrine, and dopamine during hospitalization; admission to ICU means that the patient was already admitted to the ICU at the time of receiving PMB; ICU, intensive care unit; APACHE II, Acute Physiology and Chronic Health Evaluation II; DM, diabetes mellitus; CR-GNB, carbapenem-resistant Gram-negative bacteria; TMP-SMZ, trimethoprim/sulfamethoxazole; NSAIDs, nonsteroidal anti-inflammatory drugs; PMB, polymyxin B; CRRT, continuous renal replacement therapy; Continuous data were represented as mean ± standard deviation (SD) or median and interquartile ranges (IQRs). Count data were presented as absolute numbers and percentages (%). Bold font indicates data with significant differences.

To exclude the influence of baseline kidney function on the PMB-associated nephrotoxicity, we analyzed the data with patients having normal renal function before PMB-based treatment. There were 131 patients with normal baseline renal function enrolled, and after using PMB-based treatment, 43 patients developed AKI. The nephrotoxicity incident rate in patients with baseline normal renal function was 32.8%, with 15 cases (34.88%) in stage R, 9 cases (20.93%) in stage I, and 19 cases (44.19%) in stage F (Figure 1).

### Risk Factors for AKI

The total number of AKI in all patients treated with PMB was 67 (28.63%). We assessed the potential risk factors for AKI development in patients using PMB. Univariate analysis showed that male gender, older age, mechanical ventilation, vasoactive agents, multi-site infections, disease of the digestive system, using furosemide (>20 mg/day), the maintenance dose of PMB, and baseline serum creatinine are related to AKI (all *p* < 0.05). There is no collinearity among variables in multivariate logistic regression analysis. No attribution was performed in the logistic regression analysis. After multivariable analysis, factors with higher risk of AKI include males [OR = 3.237 (1.426–7.350), *p* = 0.005], digestive system diseases [OR = 2.481 (1.127–5.463), *p* = 0.024], using furosemide (>20 mg/day) [OR = 2.473 (1.102–5.551), *p* = 0.028], and baseline serum creatinine [OR = 0.994 (0.990–0.999), *p* = 0.008] (Table 2).

When we excluded the patients with AKD or CKD before using PMB, univariate analysis showed no difference between the nephrotoxicity group and non-nephrotoxicity group on variables such as age, gender, and details of PMB regimens (all *p* > 0.05). Multivariable analysis showed that digestive system diseases are related to AKI [OR = 2.899 (1.016–8.270), *p* = 0.038] (Table 3).

**TABLE 3:** Univariate analysis and multivariate logistic regression of patients with normal renal function develop nephrotoxicity after PMB treatment.

Variable	Univariate analysis			Multivariable analysis	
	AKI [+] (*N* = 43)	AKI [-] (*N* = 88)	*p*-value	Adjusted OR (95%CI)	*p*-value
Male gender	34 (79.07)	58 (65.90)	0.122		
Age (years)	59.26 ± 16.08	55.86 ± 14.76	0.233		
Weight (kg)	60 (53–60)	60 (50–60)	0.962		
Mechanical ventilation	33 (76.74)	62 (70.45)	0.449		
Vasoactive agents	25 (58.14)	43 (48.86)	0.318		
Admission to ICU	31 (72.09)	67 (76.13)	0.617		
APACHE II	15.14 ± 6.99	18.57 ± 8.24	0.118		
Multi-site infection	23 (53.49)	38 (43.18)	0.267		
Site of infection					
*Respiratory tract*	41 (95.35)	82 (93.18)	0.922		
*Urinary system*	4 (9.30)	9 (10.22)	1.000		
*Bloodstream*	14 (32.56)	20 (22.73)	0.228		
*Abdomen*	4 (9.30)	10 (11.36)	0.954		
Underlying disease					
*Cardiovascular and cerebrovascular diseases*	22 (51.16)	51 (57.95)	0.462		
*Respiratory disease*	40 (93.02)	72 (81.82)	0.087	2.820 (0.744–10.695)	0.127
*Kidney disease*	12 (27.91)	27 (30.68)	0.794		
*Liver disease*	15 (34.88)	24 (27.27)	0.371		
*Diseases of digestive system*	12 (27.91)	13 (14.77)	0.072	**2.783 (1.057–7.327)**	**0.038**
*Nutritional diseases*	6 (13.95)	9 (10.23)	0.736		
*T2DM*	6 (13.95)	12 (13.64)	0.961		
Concomitant nephrotoxic agents	18 (41.86)	36 (40.91)	0.917		
Total number of combined nephrotoxic agents	0 (0–1)	0 (0–1)	0.973		
*Without combined nephrotoxic agents*	25 (58.14)	52 (59.09)	0.917		
*Combined with 1 nephrotoxic agent*	15 (34.88)	29 (32.95)	0.826		
*Combined with 2 nephrotoxic agents*	3 (6.97)	5 (5.68)	1.000		
*Combined with 3 nephrotoxic agents*	0 (0)	2 (2.27)	0.812		
*Vancomycin*	0 (0)	9 (10.23)	0.071	-	0.999
*Aminoglycosides*	0 (0)	4 (4.55)	0.379		
*Amphotericin B*	1 (2.33)	4 (4.55)	0.891		
*TMP-SMZ*	4 (9.30)	4 (4.55)	0.497		
*Fluorocytosine*	0 (0)	1 (1.14)	1.000		
*Furosemide* (>*20 mg/day*)	11 (25.58)	12 (13.64)	0.092	2.094 (0.797–5.504)	0.134
*Torasemide* (>*10 mg/day*)	2 (4.65)	5 (5.68)	1.000		
*NSAIDs*	2 (4.65)	5 (5.68)	1.000		
*Ciclosporin*	1 (2.33)	1 (1.14)	0.550		
Pre-medication kidney disease*					
*CRRT*	1 (2%)	0% (0%)	0.727		
*Hemodialysis*	0 (0%)	0 (0%)	-		
Antimicrobial susceptibility					
Polymyxin MIC >2 (mg/L)	1 (2.33)	4 (4.55)	0.891		
*Applied load dose*	25 (58.13)	42 (47.73)	0.263		
*Loading dose in mg/kg*	1.44 ± 0.56	1.35 ± 0.57	0.282		
*Maintenance dose in mg/kg(q12 h)*	0.96 ± 0.22	0.92 ± 0.24	0.254		
*Meet the minimum standard load dose*	11 (25.58)	20 (22.73)	0.718		
*Minimum maintenance dose achieved*	6 (13.95)	10 (11.36)	0.670		
*Cumulative dose*	1,050 (550–1,650)	980 (600–1,300)	0.444		
*Treatment duration time (days)*	10 (5.75–13)	9 (6–12.5)	0.720		
*Maintenance dose q12 h (mg)*	50 (50–50)	50 (50–50)	0.135		
*Loading dose (mg)*	100 (50–100)	50 (50–100)	0.267		
*Baseline serum creatinine (umol/L)*	46.90 (35.90–64.40)	55.20 (40.58–71.18)	0.219		

AKI, acute kidney injury; vasoactive agents mainly contain epinephrine, norepinephrine, isopropyl epinephrine, and dopamine during hospitalization; admission to ICU means that the patient was already admitted to the ICU at the time of receiving PMB; ICU, intensive care unit; APACHE II, Acute Physiology and Chronic Health Evaluation II; DM, diabetes mellitus; CRO, carbapenem-resistant organisms; TMP-SMZ, trimethoprim/sulfamethoxazole; NSAIDs, nonsteroidal anti-inflammatory drugs; PMB, polymyxin B; CRRT, continuous renal replacement therapy; Continuous data were represented as mean ± standard deviation (SD) or median and interquartile ranges (IQRs). Count data were presented as absolute numbers and percentages (%). Bold font indicates data with significant differences.

### Risk Factors of AKI Severity

**FIGURE 2:**
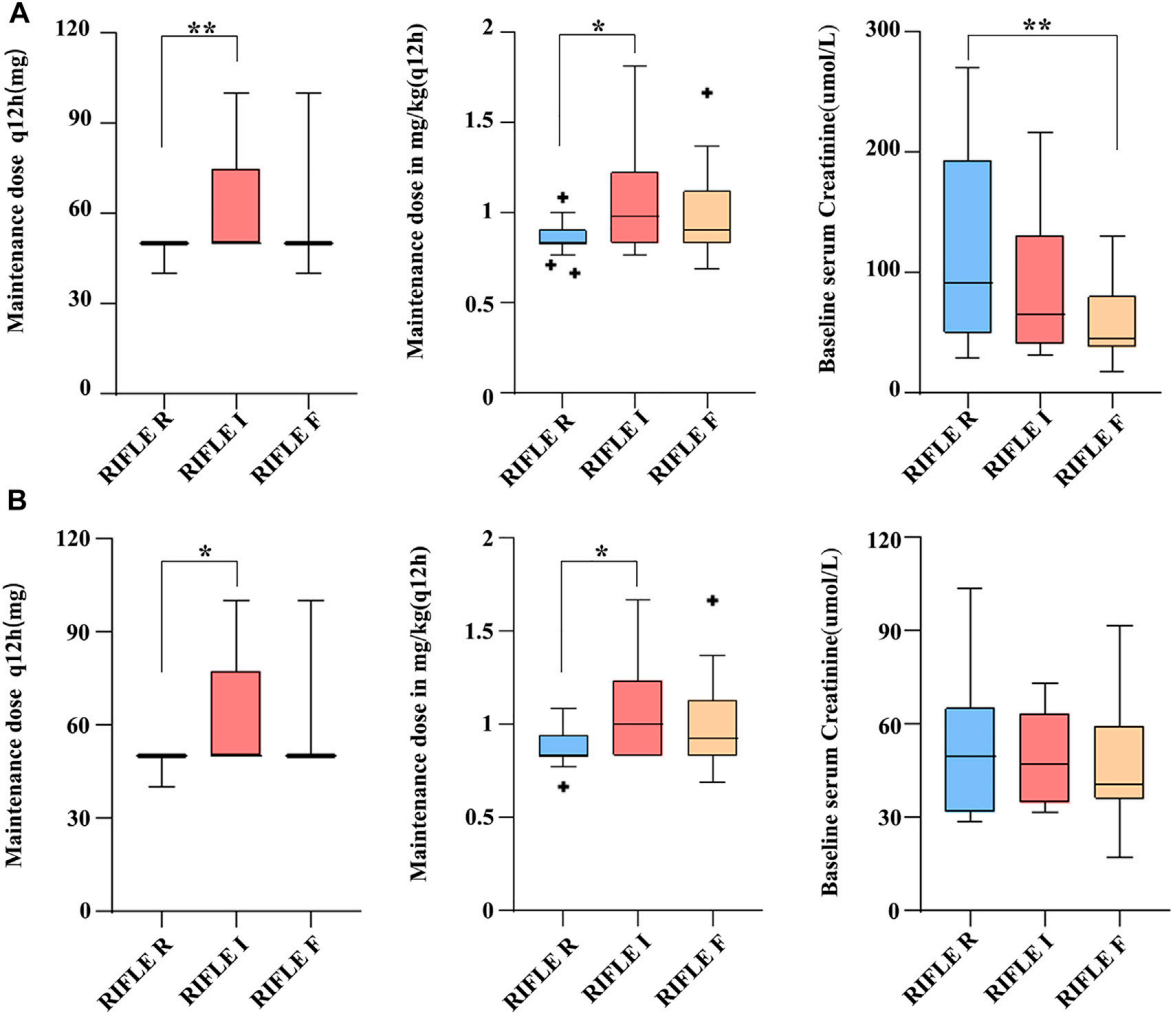
Comparisons of baseline serum creatinine and PMB dose among patients with PMB-associated AKI according to different RIFLE stages. ∗*p* < 0.05, **p < 0.005. **(A)** All patients having AKI and **(B)** patients having AKI with normal renal function before PMB regimen.

Nonparametric tests of K-independent samples were used to explore the risk factors related to the severity of AKI in patients using PMB. We found that there were significant differences among different AKI stages in baseline serum creatinine, the maintenance dose of PMB (mg), and the maintenance dose (mg/kg) (all *p* < 0.05). In the pairwise comparison by univariate analysis, we found that baseline serum creatinine is higher in the RIFLE R group than that in the RIFLE F group [91 (49.7–193.5) vs. 44.5 (37.7–80.85) μmol/L, *p* = 0.003]. The maintenance dose of PMB is higher in the RIFLE I group than that in the RIFLE R group [50 (50–70) vs. 50 (50–50) mg/q12 h, *p* = 0.001; 0.980 (0.833–1.231) vs. 0.833 (0.833–0.909) mg/kg/q12 h, *p* = 0.023] (Table 4 and Figure 2).

**TABLE 4:** Factors potentially associated with the RIFLE classification of acute kidney injury in all patients treated with PMB.

Characteristic	RIFLE R	RIFLE I	RIFLE F	*p*-value	P_1_	P_2_	P_3_
	*N* = 31	*N* = 15	*N* = 21				
*Baseline serum creatinine(μmol/L)*	91.00 (49.70–193.50)	65.20 (40.50–65.20)	44.50 (37.70–80.85)	**0.010**	0.223	**0.003**	0.112
*Maintenance dose q12 h(mg)*	50.00 (50.00–50.00)	50.00 (50.00–75.00)	50.00 (50.00–50.00)	**0.009**	**0.001**	0.145	0.157
*Maintenance dose in mg/kg(q12 h)*	0.833 (0.833–0.909)	0.980 (0.833–1.230)	0.909 (0.833–1.124)	**0.042**	**0.023**	0.062	0.582

RIFLE (Risk, Injury, Failure, Loss of kidney function, and End-stage renal disease) criteria. *p*-value, the comparison of three groups; P_1_, RIFLE R vs. RIFLE I; P_2,_ RIFLE R vs. RIFLE F; P_3,_ RIFLE I vs. RIFLE F. Bold font indicates data with significant differences.

When we excluded the patients with AKD and CKD before using PMB, we found significant differences among different AKI stages in the maintenance dose (mg/q12 h) of PMB (*p* = 0.016). In the pairwise comparison by univariate analysis, we found that the maintenance dose of PMB is higher in the RIFLE I group than that in the RIFLE R group [50.00 (50.00–75.00) vs. 50.00 (50.00–50.00) mg/q12 h, *p* = 0.006; 1.000 (0.833–1.240) vs. 0.833 (0.833–0.943) mg/kg/q12 h, *p* = 0.04] (Table 5 and Figure 2).

**TABLE 5:** Univariate analysis of patients with normal renal function developing nephrotoxicity (staged using the RIFLE classification) after PMB treatment.

Characteristic	RIFLE R	RIFLE I	RIFLE F	*p*-value	P_1_	P_2_	P_3_
	*N* = 15	*N* = 9	*N* = 19				
*Baseline serum creatinine(μmol/L)*	49.70 (31.5–65.30)	46.90 (34.55–63.55)	40.40 (35.90–59.90)	0.763	0.811	0.456	0.749
*Maintenance dose q12 h(mg)*	50.00 (50.00–50.00)	50.00 (50.00–77.50)	50.00 (50.00–50.00)	**0.016**	**0.006**	0.059	0.145
*Maintenance dose in mg/kg(q12 h)*	0.833 (0.833–0.943)	1.000 (0.833–1.240)	0.926 (0.833–1.136)	0.138	**0.040**	0.244	0.345

RIFLE (Risk, Injury, Failure, Loss of kidney function, and End-stage renal disease) criteria. *p*-value, the comparison of three groups; P_1_, RIFLE R vs. RIFLE I; P_2,_ RIFLE R vs. RIFLE F; P_3,_ RIFLE I vs. RIFLE F. Bold font indicates data with significant differences.

## Discussion

We conducted a retrospective cohort study about PMB-associated nephrotoxicity and its predictors. Our study found that the AKI incidence was 28.63% in 234 CR-GNB-infected patients with PMB-based regimens. Males, digestive system diseases, using furosemide (>20 mg/day), and high baseline serum creatinine were associated with a higher risk for AKI during hospitalization. Excluding patients with AKD or CKD before PMB use, digestive system diseases and furosemide (>20 mg/day) were associated with a high risk for AKI. The severity of acute kidney injury is related to the maintenance dose of PMB and high baseline serum creatinine. These risk factors should be taken into consideration when initiating PMB-based therapy.

The incidence rate of PMB-related nephrotoxicity ranges from 11.8 to 50.6% in previous studies (Nelson et al., 2015; John et al., 2017; Soares et al., 2017; Aggarwal and Dewan, 2018; Cai et al., 2020; Sisay et al., 2020; Zhang et al., 2021). Our study subjects comprise a Chinese population. Whether the ethnics contribute to the incidence rate of PMB-related nephrotoxicity is still not known. The AKI rate in our study was 28.63%, which was lower than the rate reported in previous reports on Caucasians and Asians (Nelson et al., 2015; John et al., 2017; Soares et al., 2017; Aggarwal and Dewan, 2018; Cai et al., 2020; Sisay et al., 2020; Zhang et al., 2021). This may be due to various reasons that lead to the high variation of AKI incidence in different studies, such as different population characteristics, renal function status, PMB doses, and the different proportions of drug use with nephrotoxicity. Patients with fluctuating renal function were not excluded, which might have increased the AKI rates (John et al., 2017). In addition, using different entry criteria may also be one of the influencing factors. The low rates of nephrotoxicity with PMB may be associated with the patient on any other nephrotoxic drug and who received significant doses of diuretics, for diuretics have shown to potentiate polymyxin nephrotoxicity (Deryke et al., 2010; Pogue et al., 2011). Similarly, in the study by Yarelis et al. (2019), the patients with a baseline creatinine clearance (CrCl) <10 ml/min and/or on renal replacement were also excluded. However, the overall rate of AKI in this cohort was 35% (Yarelis et al., 2019). In this cohort, the dosing for PMB ranged from 15,000 to 25,000 U/kg/day (Yarelis et al., 2019). In our study, 79.06% of patients used the low daily doses of PMB compared to the recommended doses, as Pooja et al. suggested that a higher daily dose of PMB was associated with a more rapid onset of nephrotoxicity (Manchandani et al., 2017). A total of 46% of patients developed any degree of AKI during PMB treatment with the high dose reported by John et al. (2017). In this study, patients received PMB at a dose of >3 mg/kg of body weight/day or a total dose of ≥250 mg/day, while the dose in our study was 1.68 (1.66–2.00) mg/kg/day. Moreover, after excluding the AKD or CKD patients before PMB use, the AKI rate in patients with normal baseline serum creatinine was 32.8%, similar to previously published data by Zhang et al. (2021) in which the AKI rate was higher with 38.7%. In their study, the daily dose of PMB is also less than 2 mg/kg. A recent study reported that 11.8% of patients’ incidence of nephrotoxicity was in the PMB group (Aggarwal and Dewan, 2018). The source of discrepancy used different criteria to define nephrotoxicity; the “risk I” category was excluded in their study, even though RIFLE criteria were used (Aggarwal and Dewan, 2018).

We also found that the maintenance dose of PMB in the AKI group is higher than in non-AKI groups (*p* = 0.048). The loading dose rate and loading dose have the trend of being higher in the AKI group [52.25% vs. 38.92%, *p* = 0.063; 80 (50–100) vs. 50 (50–100) mg, *p* = 0.057]. Therefore, the doses of PMB are associated with the incidence of AKI. Our study results are consistent with previous meta-analyses (Oliota et al., 2019; Sisay et al., 2020; Wagenlehner et al., 2020) and case–control studies (Cai et al., 2020; Zhang et al., 2021). A recent meta-analysis found that older age, the daily dose [OR = 1.46 (1.09–1.96)], underlying diabetes mellitus, and concomitant nephrotoxic drugs were independent risk factors for polymyxin-induced nephrotoxicity (Sisay et al., 2020).

Moreover, we first report that the maintenance dose of PMB is also associated with the severity of AKI. In the univariate analysis of the pairwise comparison of AKI severity, we found that between the R stage and I stage, the greater the maintenance dose of PMB, the greater the severity of AKI risk. However, the maintenance dose of PMB did not show a difference in stage F, which may indicate that PMB does not cause serious kidney damage. The maintenance dose of PMB in this study is lower than the standard dose of PMB (1.25–1.5 mg/kg q12 h) and does not cause severe AKI. Larger prospective studies on the dose of PMB to nephrotoxicity are needed.

Our data showed that the independent risk factors associated with AKI development included males, digestive system diseases, using furosemide, and high baseline serum creatinine. In the study by Sisay et al. (2020), though they were not statistically significant, they were 1.52 times more likely to induce nephrotoxicity. We observed that males were one risk factor of AKI. Sisay et al. (2020)also reported that the PMB-associated nephrotoxicity in male patients is 1.52 times more than that in female patients. The previous study also implied that the male sex is associated with an increased incidence of hospital-associated AKI requiring renal replacement therapy (Neugarten et al., 2018). They suggested that reno-protection is mediated by the effects of sex hormones on cellular processes instrumental in the pathogenesis of AKI, analogous to our suggestion that sex hormones mediate the beneficial effects of female sex on the course of chronic kidney disease (Neugarten et al., 2018). Sex-related differences in the generation of nitric oxide, in the synthesis and vascular response to endothelin-1, in the inflammatory, hemodynamic, and humoral responses to ischemia–reperfusion injury, and in the renal hemodynamic response to angiotensin II have been demonstrated in experimental models and human subjects (Takayama et al., 2007; Hutchens et al., 2008). Whether these sex-related differences influence the incidence of PMB-associated AKI needs further research.

Older age is a known risk factor for AKI that has also been found in previous studies with PMB (Balkan et al., 2014; Phe et al., 2014). Although the median age of the AKI group was higher than that of the non-AKI group, there was no difference in the older age in our study. Using furosemide is also associated with the onset of nephrotoxicity in our research. Diuretics have potentiated polymyxin-associated nephrotoxicity in previous studies (Deryke et al., 2010; Pogue et al., 2011). In our study, digestive system diseases were one of the risk factors for AKI. Alkhatib et al (2009) demonstrated that the prevalence of AKI is higher in elderly patients presenting with acute upper gastrointestinal bleeding (48.7%). Zhang et al. (2008) proposed that TNF-α acts directly on glomeruli and renal tubular capillaries, leading to ischemia and tubular necrosis. This may increase the platelet-activating factor, inducing and producing inflammatory mediators (Zhang et al., 2008). The secretion of phospholipase A2 (PLA_2_), which originates from platelets, can produce a large quantity of thromboxane A2 (TXA_2_) and prostacyclin (PGI_2_). These two substances function in angiotasis regulation (Zhang et al., 2008). The proportional imbalance of TXA2/PGI2 can cause vasomotion disturbance, the formation of microthrombus, vascular occlusion, and other pathological changes, leading to abnormal contraction of blood vessels of the kidney, a decline in renal blood flow, and perfusion of kidney tissue (Zhang et al., 2008). These changes can cause serious injury to the kidney. Inflammatory mediators may increase mucosal permeability, causing endotoxins and bacteria to translocate from the colon. Endotoxin increases the level of endothelin, leading to vasoconstriction, renal blood flow reduction, and renal tubular necrosis, thereby promoting the development of AKI (Bose et al., 2002). Zhang et al. (2018) stated that during sepsis, the combined effect of erosion of the mucus barrier, a shift in the composition and virulence of intestinal microbes, and the inability of the host epithelium to regulate its proliferative and apoptotic responses might lead to a tipping point in gut function in which cascading inflammation drives AKI. The potent direct nephrotoxic effects of polymyxins include mechanisms that kill bacteria *via* interactions with lipid A, disrupting the Ca^2+^ and Mg^2+^ bridges, which destabilize the lipopolysaccharide molecules in the bacterial membrane, and the effects on D-amino content and fatty acid components that increase membrane permeability and the influx of cations (Falagas and Kasiakou, 2006; Velkov et al., 2013). In both LLC-PK1 cells and rat kidney models, PMB reduced creatinine clearance. It increased renal vascular resistance and oxidative damage, demonstrating that PMB nephrotoxicity is characterized by mitochondrial dysfunction and free radical generation (Vattimo Mde et al., 2016). These factors, such as males, digestive system diseases, using furosemide, and high baseline serum creatinine, increase the risk of PMB-associated AKI incidence and need further mechanistic research.

In the rate of AKI, the baseline serum creatinine is connected with AKI before we exclude the AKD or CKD patients. Moreover, the lower baseline serum creatinine has a higher risk of AKI. This is similar to the study by Aggarwal and Dewan (2018), which found that patients with low baseline creatinine clearance had a significantly lower incidence of renal failure than patients with high creatinine clearance.

One of the strengths of our study was that we included 234 patients, which is relatively large sample size data compared to recent studies about PMB-associated nephrotoxicity in the Chinese population. We strictly followed KDGIO’s definition of AKI and used the RIFLE classification to standardize the severity of AKI. Our study was limited by not eliminating all possible confounding factors contributing to renal failure. There is no general laboratory data such as complete blood count, liver function test, and infection markers such as C-reactive protein, which is also the limitation of our study. Moreover, due to the complexity of the causes of AKI, many possible related factors, such as other drugs used at the same time, other underlying diseases, and other factors that may affect the occurrence of AKI, were not considered in the study, which is also the limitation of our study. This project is a single-center retrospective cohort study, which requires further verification by a multi-center prospective cohort study with a larger scale size.

In conclusion, our study demonstrated that male, digestive system diseases, using furosemide (>20 mg/day), and high baseline serum creatinine were the independent risk factors of PMB-associated AKI development. The serum creatinine value should be closely monitored when using PMB. The maintenance dose of PMB may be related to the severity of AKI, and these risk factors should be considered when initiating PMB-based therapy.

## Data Availability

The original contributions presented in the study are included in the article/Supplementary Material, further inquiries can be directed to the corresponding author.

## Abbreviations

ADRs, adverse reactions; AKI, acute kidney injury; AKD, acute kidney disease; CKD, chronic kidney disease; CR-GNB, carbapenem-resistant Gram-negative bacteria; IQR, interquartile range; KDIGO, kidney disease: improving global outcomes; MDR, multi-drug resistance; SD, standard deviation; TMP-SMZ, trimethoprim/sulfamethoxazole.
